# Impact of adenosine on mechanisms sustaining persistent atrial fibrillation: Analysis of contact electrograms and non-invasive ECGI mapping data

**DOI:** 10.1371/journal.pone.0248951

**Published:** 2021-03-25

**Authors:** Gurpreet Singh Dhillon, Nikhil Ahluwalia, Shohreh Honarbakhsh, Adam Graham, Antonio Creta, Hakam Abbass, Anthony Chow, Mark J. Earley, Pier D. Lambiase, Richard J. Schilling, Ross J. Hunter

**Affiliations:** Barts Heart Centre, St Bartholomew’s Hospital, Barts Health NHS Trust, London, United Kingdom; Kurume University School of Medicine, JAPAN

## Abstract

**Background:**

We evaluated the effect of adenosine upon mechanisms sustaining persistent AF through analysis of contact electrograms and ECGI mapping.

**Methods:**

Persistent AF patients undergoing catheter ablation were included. ECGI maps and cycle length (CL) measurements were recorded in the left and right atrial appendages and repeated following boluses of 18 mg of intravenous adenosine. Potential drivers (PDs) were defined as focal or rotational activations completing ≥ 1.5 revolutions. Distribution of PDs was assessed using an 18 segment biatrial model.

**Results:**

46 patients were enrolled. Mean age was 63.4 ± 9.8 years with 33 (72%) being male. There was no significant difference in the number of PDs recorded at baseline compared to adenosine (42.1 ± 15.2 vs 40.4 ± 13.0; p = 0.417), nor in the number of segments harbouring PDs, (13 (11–14) vs 12 (10–14); p = 0.169). There was a significantly higher percentage of PDs that were focal in the adenosine maps (36.2 ± 15.2 vs 32.2 ± 14.4; p < 0.001). There was a significant shortening of CL in the adenosine maps compared to baseline which was more marked in the right atrium than left atrium (176.7 ± 34.7 vs 149.9 ± 27.7 ms; p < 0.001 and 165.6 ± 31.7 vs 148.3 ± 28.4 ms; p = 0.003).

**Conclusion:**

Adenosine led to a small but significant shortening of CL which was more marked in the right than left atrium and may relate to shortening of refractory periods rather than an increase in driver burden or distribution.

Registered on Clinicaltrials.gov: NCT03394404.

## Introduction

The effects of Adenosine upon mechanisms sustaining persistent Atrial Fibrillation (AF) are not well understood. Persistent AF is thought to be maintained by localised sources termed drivers that are intermittent but recur at patient specific sites [1–4]. There is now increasing interest in identifying and targeting these drivers using contact and non-contact mapping during catheter ablation procedures [1, 2, 5].

Adenosine is an endogenous nucleoside commonly used to diagnose and treat supraventricular tachycardias [6]. Adenosine shortens atrial action potential duration and refractory periods [7]. In catheter ablation procedures for AF, adenosine is used to unmask dormant pulmonary vein conduction post pulmonary vein isolation [8, 9]. Studies utilizing adenosine in this respect have also observed an increase in PV firing during administration [10]. There is therefore potential for adenosine to affect both focal and reentrant mechanisms in AF. The impact of adenosine on atrial repolarization and refractoriness may be greater in the right compared to the left atrium, although why this should be is unclear [11]. Regional differences in expression of adenosine sensitive receptors such as adenosine A1 may explain this heterogenous effect and may cause site specific effects on AF mechanisms [10, 11]. Greater understanding of the effect of adenosine may improve our understanding of AF mechanisms generally, but are also important for clinical and research purposes. Electrocardiographic Imaging (ECGI) mapping and non-contact mapping have both been used to study mechanisms in AF but also often require adenosine administration to produce pauses without QRS complexes for analysis, which then has an undetermined impact on AF mechanisms.

We hypothesized that Adenosine would have a significant impact on AF mechanisms. We explored this through contact electrograms to examine left and right atrial appendage (LAA and RAA) cycle length (CL) in addition to ECGI mapping to examine the burden and distribution of focal and rotational activations before and after administration of adenosine.

## Methods

### Patient population

**Fig 1:**
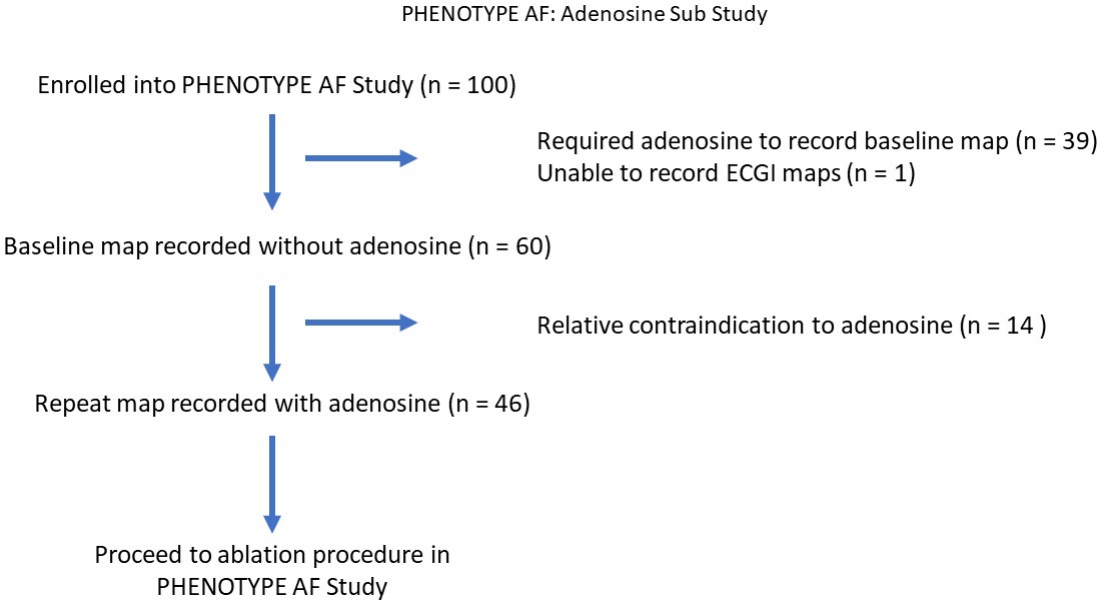
Flowchart of phenotype AF: Adenosine sub study. Flowchart describing how patients enrolled into the Phenotype AF study were recruited to this sub study.

This study is a sub-study of a clinical trial registered on clinicaltrials.gov (NCT03394404). Approved by East Midlands—Leicester South Research Ethics Committee REC reference: 17/EM/0333, IRAS project ID:218367. Patients undergoing first time catheter ablation for persistent AF of less than two years duration were prospectively enrolled between January and December 2018 (Fig 1). All participants provided written informed consent. Exclusion criteria included: LA diameter > 5cm, LV EF < 40%, NYHA III or IV heart failure, age < 18 or > 80 years, hypertrophic cardiomyopathy or greater than moderate valve disease.

### Non-invasive ECGI mapping

Patients were fitted with the ECGI multi-electrode vest. They then underwent a non-contrast computed topography (CT) scan. A 3D bi-atrial geometry was then manually generated from the CT scan on the ECGI computer system (CardioInsight, Medtronic, USA). The ECGI system is then able to determine the position of the surface electrodes and the surface of the heart.

All ECGI mapping was performed intra-procedurally prior to any ablation. If patients were in sinus rhythm then AF was induced through pacing and left to stabilize for at least 10 minutes prior to mapping. 15 seconds of cumulated atrial intervals, each of a minimum duration of ≥ 840 ms were collected to generate a bi-atrial map of potential drivers (PDs). Intravenous beta-blockers or calcium channel blockers were administered if the ventricular rate required slowing. After 15 seconds of data had been collected for a map, adenosine was then administered in 18 mg boluses to collect a further 15 seconds of data for a separate post adenosine map. As it was unclear whether adenosine would elicit a response detectable with the technologies utilized, a single large dose was studied rather than a complex dose response relationship. A dose of 18 mg was chosen as this is commonly employed to slow the ventricular rate to allow ECGI mapping, and hence it is particularly important to determine whether a dose in this range impacts atrial electrophysiology significantly [5]. Potential drivers (PDs) were identified based on ECGI mapping and were defined using similar definitions to previous work from our institution and others [1, 5, 12]. PDs were defined as either focal activations or rotational activations completing at least 1.5 revolutions.

### Offline analysis

**Fig 2:**
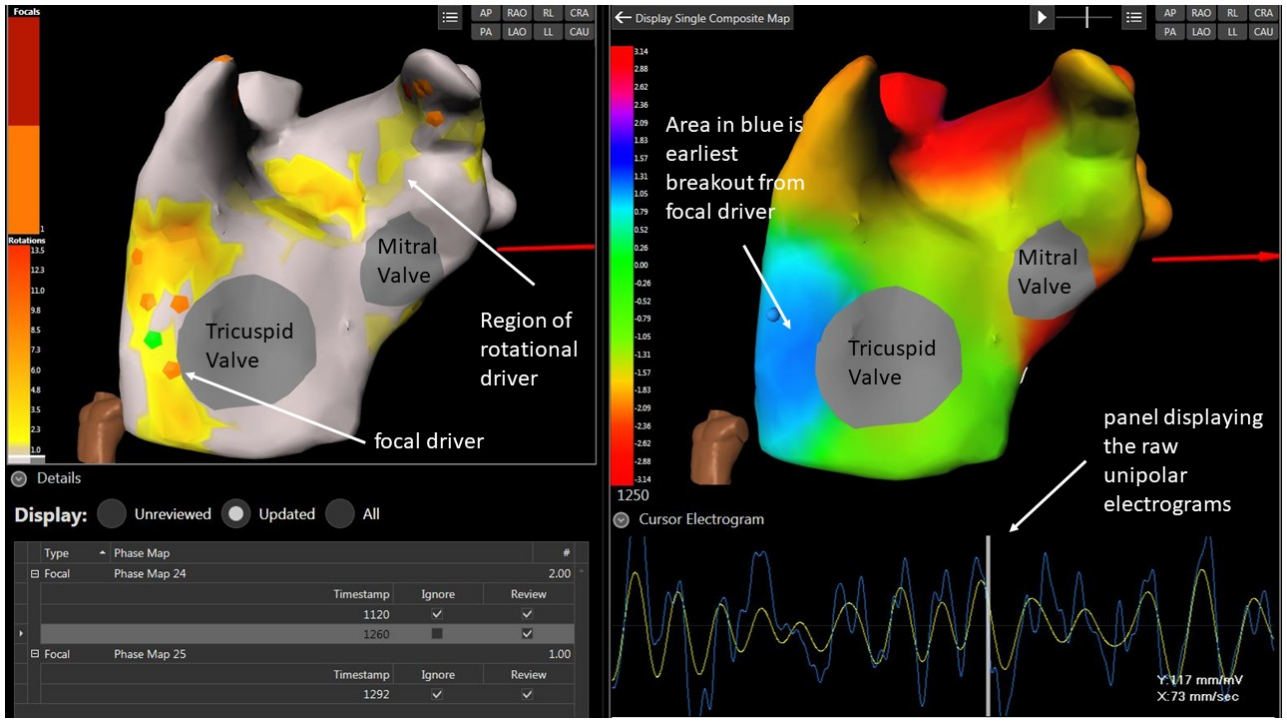
Image from the ECGI system showing a focal activation being reviewed. Screenshot from the ECGI workstation software. This screen allows the operator to select any potential focal or rotational driver and for review. Far left: LAO view of the composite biatrial map displaying focal PDs (orange hexagons) and yellow/orange areas as a heat map with darker colour showing greater number of rotational PDs occurrences. A selected focal PD is highlighted in green. Right image: a phase map with the activation sequence for that focal PD with the blue region showing the site of earliest activation, spreading out through light blue, green, then yellow and red colours. The raw unipolar electrogram is displayed on the bottom right panel. The software can display the unipolar electrograms from any point on the phase map.

Offline analysis was performed post procedure by 2 operators blinded to which map was with adenosine or at baseline. Firstly, operators would review the surface ECG recordings and atrial segments with excessive noise would be excluded. Excess segments were collected during procedures to ensure that the final ECGI map comprised at least 15000 ms. Secondly, the raw unipolar electrograms were reviewed. Individual electrodes from the ECGI jacket that exhibited excessive noise were removed. Finally, the individual PDs were displayed on a biatrial composite map and individually reviewed (Fig 2). If the PD appeared to be implausible, they were then excluded.

### Potential driver data analysis

PDs were assessed in terms of burden and characteristics: the total number of PD occurrences (including rotational and focal occurrences), sum of revolutions and repetitive activations of focal PDs, the stability of rotational activation patterns (the mean number of revolutions per rotational PD occurrence), the proportion of PDs that were rotational or focal. PDs were also assessed on a distribution basis using a bespoke 18 segment model described previously [12]. The number of segments harbouring drivers at the PVs and posterior wall and elsewhere, segments harbouring drivers in the RA, LA, LA excluding PVs and posterior wall, and septum were collected. Where a PD occupied an area that straddled more than one segment on the 18-segment model, it was counted as a single driver occurrence but ascribed to more than one segment for the purposes of assessing distribution.

### Contact mapping acquisition

**Fig 3:**
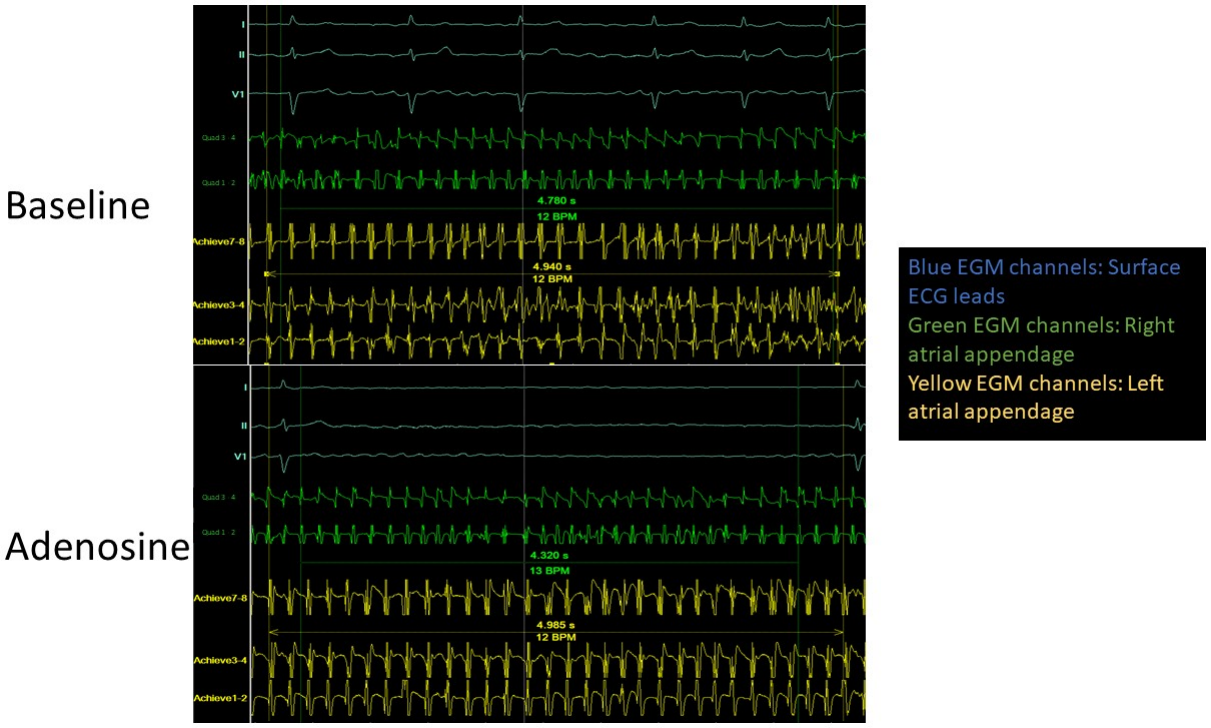
Cycle length measurements at baseline and with adenosine. Cycle length measurements from the same patient at baseline and with Adenosine. The surface ECG leads are shown at the top (blue channels) with a quadripolar catheter in the right atrial appendage (green channels) and the Achieve mapping catheter in the left atrial appendage (yellow channels). Cycle length recordings taken in this patient at the RAA are shorter in the Adenosine maps compared to baseline but are similar in both at the LAA.

LabSystem Pro (Boston Scientific, Marlborough, MA, USA) was used to record and display electrogram data. A quadripolar catheter was sited at the RAA and a circular mapping catheter was sited at the LAA in order to record cycle lengths over 30 cycles during acquisition of the ECGI maps before and after administration of adenosine, Fig 3.

### Study end points

The co-primary end points were the impact of adenosine on PD burden (defined as the number of driver occurrences) and distribution (defined as the number of segments harbouring drivers on the 18-segment model). Secondary end points included an assessment of the impact adenosine on PD temporal stability, and the proportion of PDs that were focal versus rotational. The impact of adenosine on LAA and RAA CL was also assessed.

### Statistical analysis

Normally-distributed data were expressed as mean ± standard deviation or if not normally-distributed as median with interquartile range. Student’s paired t test was performed for normally-distributed variables and Wilcoxon paired samples test was performed for non-parametric variables. A multivariate analysis was performed using binary logistic regression to determine if there were predictors of RAA or LAA reduction or change in total PD burden or PD distribution. A change of 15% was thought to be clinically significant and taken as a positive response. Factors included as categorical covariates included gender, hypertension, diabetes mellitus and ischaemic heart disease. Continuous factors included age and LA diameter. Factors were removed from the model in a stepwise fashion until only factors with a p-value of < 0.10 remained in the final model. Spearman’s rank correlation was used to determine correlation between either AF duration or LA diameter with change in RAA CL, LAA CL, PD burden and PD distribution with adenosine administration. Statistical analysis were performed using SPSS (IBM SPSS Statistics, Version 25 IBM Corp, Armonk, NY, USA). A P-value of <0.05 was taken to indicate statistical significance. Power calculations were performed using G*Power 3.1 (G*Power, version 3.1.9.6, Heinrich-Heine-Universität Düsseldorf, Germany) [13].

## Results

In total 46 patients were included in this study from 100 who were enrolled into the Phenotype AF study (Fig 1). Patient demographic data is displayed in Table 1. Mean age was 63.4 ± 9.8 years with 33 (72%) being male. Mean LA diameter was 39.6 ± 5.9 millimetres (mm) with median time from diagnosis of AF to ablation being 18 (11–31) months with median duration of AF being 12 (6–17) months. ECGI maps at baseline and with adenosine were generated in all patients (a total of 92 maps were analysed).

**Table 1:** Participant demographics.

Baseline Characteristics	
Number of Patients	46
Age (years) Mean ± SD	63.4 ± 9.8
Male n (%)	33 (72)
Hypertension n (%)	20 (43)
Diabetes Mellitus n (%)	10 (22)
Ischaemic Heart Disease n (%)	6 (13)
Cerebrovascular Accident n (%)	3 (7)
CHA2DS2VASC Score mean ± SD	2.0 ± 1.5
LA Diameter (mm) ± SD	39.6 ± 5.9
LA Volume (ml) ± SD	68.7 ± 23.0
Median diagnosis of AF to procedure (months)	18 (11–31)
Duration of Persistent AF (months)	12 (6–17)
Persistent AF (< twelve months)	25
Longstanding AF (> twelve months)	21

Values are given as no. (%), mean ± standard deviation or median (Interquartile Range).

### Baseline MAPS

46 atrial maps were generated. The median duration of ECGI recordings per map was 15.2 (15.1–15.6) seconds which was comprised of 16 (15–16) intervals. The total number of PD occurrences was 42.1 ± 15.2. On a regional analysis, the number of PD occurrences at the pulmonary veins and posterior wall (PVs and PW) was 9.5 ± 5.0, and the number occurring outside the PVs and PW was 32.5 ± 13.1.

The number of segments on the 18 segment model harbouring drivers was 13 (11–14). On a regional analysis, the number of segments harbouring drivers at the PVs and PW was 4 out of 5 segments (3–4), and the number of segments outside the PVs and PW was 9 out of 13 (8–10).

### Impact of adenosine

Although there was no significant difference in the cumulative duration of ECG segments for analysis (15.2 (15.1–15.6) s at baseline versus 15.5 (15.2–15.5) s post adenosine; p = 0.797) the number of intervals recorded was significantly fewer with the adenosine derived maps as expected (16 (15–16) intervals for baseline maps vs 10 (9–12) intervals post adenosine, p <0.001).

#### i) Impact on burden of PDs

The burden and characteristics of PDs recorded in baseline and adenosine derived maps are shown Table 2. Comparison of the total number of PD occurrences recorded at baseline (42.1 ± 15.2) and with adenosine (40.4 ± 13.0) did not reveal any significant difference; p = 0.417. Nor was there a significant difference on a regional basis with number of PDs recorded at the PVs and PW (9.5 ± 5.0 recorded at baseline compared to 9.0 ± 4.2 with adenosine; p = 0.399) and elsewhere excluding the PVs and PW (32.5 ± 13.1 at baseline versus 31.5 ± 11.2 with adenosine; p = 0.554).

**Table 2:** Comparison of potential driver burden in ECGI maps at baseline compared to those with adenosine.

Factor	Baseline ECGI Maps	Adenosine ECGI Maps	P Value
No of PDs Occurrences at PVs and PW	9.5 ± 5.0	9.0 ± 4.2	0.399
No of PDs Occurrences (excluding those at the PVs and PW)	32.5 ± 13.1	31.5 ± 11.2	0.554
Total No of PD Occurrences	42.1 ± 15.2	40.4 ± 13.0	0.417
Sum of Rotations at the PVs and PW	12.4 ± 8.9	11.1 ± 7.5	0.305
Sum of Rotations exc. those at the PVs and PW	54.7 ± 26.1	55.5 ± 25.9	0.825
Total Sum of Rotations	67.1 ± 31.2	66.6 ± 30.1	0.914
Sum of Foci at the PVs and PW	4.3 ± 3.4	4.9 ± 3.6	0.275
Sum of Foci exc. those at the PVs and PW	8.6 ± 4.8	9.0 ± 3.8	0.627
Total Sum of Foci	12.9 ± 6.4	13.9 ± 5.4	0.296
Percent of PDs that were Focal	32.2 ± 14.4	36.2 ± 15.2	<0.001
Mean no of Rotations (per PD occurrence)	2.3 ± 0.4	2.3 ± 0.3	0.773
Proportion of PD occurrences at PV and PW	0.3 ± 0.2	0.3 ± 0.2	0.700

Values given as mean ± standard deviation or median (interquartile range). Number of PD occurrences is the total sum of the total number of focal and rotational PD occurrences. Sum of rotations is the sum of revolutions of the all the accumulated rotational PDs.

#### ii) Impact upon potential driver distribution

No significant difference was seen in the number of segments harbouring PDs between the maps at baseline and those derived with adenosine (13 (11–14) vs 12 (10–14); p = 0.169), nor were there any regional difference when comparing segments at the PVs and PW (4 (3–4) vs 3 (3–4); p = 0.215) or segments excluding those at the PVs and PW (9 (8–10) vs 9 (8–10); p = 0.367) (Table 3). Comparison of percentage of segments harbouring PDs in the LA, septum and RA did not reveal any significance difference between the PD maps (p > 0.325).

**Table 3:** Comparison of potential driver distribution in maps with adenosine and without.

Factor	Baseline	Adenosine	P Value
No of Segments harbouring PDs at PVs and PW	4 (3–4)	3 (3–4)	0.215
No of Segments (excluding those at the PVs and PW)	9 (8–10)	9 (8–10)	0.367
Total No of Segments harbouring PDs	13 (11–14)	12 (10–14)	0.169
Proportion of PDs at the PV and Posterior Wall to Elsewhere	0.41 ± 0.16	0.40 ± 0.15	0.615
Percentage of Segments harbouring PDs in the LA	59.2 ± 7.3	57.8 ± 7.2	0.325
Percentage of Segments harbouring PDs at Septum	12.9 ± 5.0	13.8 ± 6.3	0.355
Percentage of Segments harbouring PDs in the RA	27.8 ± 7.3	28.4 ± 7.2	0.633

Distribution is described as the number of segments of the atria on an 18 segment model that harboured PDs. Distribution in certain regions is also analysed. Pulmonary veins and posterior wall are abbreviated to PVs and PW. LA is left atrium and RA is right atrium. Values given as mean ± standard deviation or median (interquartile range).

#### iii) Impact on PD characteristics and stability

No significant difference was seen in the sum of revolutions of the rotational PDs (67.1 ± 31.2 vs 66.6 ± 30.1; p = 0.914) nor was there any difference in the sum of the focal PDs (12.9 ± 6.4 vs 13.9 ± 5.4; p = 0.296). Comparison of regional difference between the sum of rotational or focal PDs at the PVs and PW, or those occurring outside the PVs and PW did not reveal any significant difference either (all p > 0.05). When comparing the stability of PDs by calculating the mean number of revolutions completed per rotational PD occurrence, no significant difference was seen (2.3 ± 0.4 vs 2.3 ± 0.3; p = 0.773).

**Fig 4:**
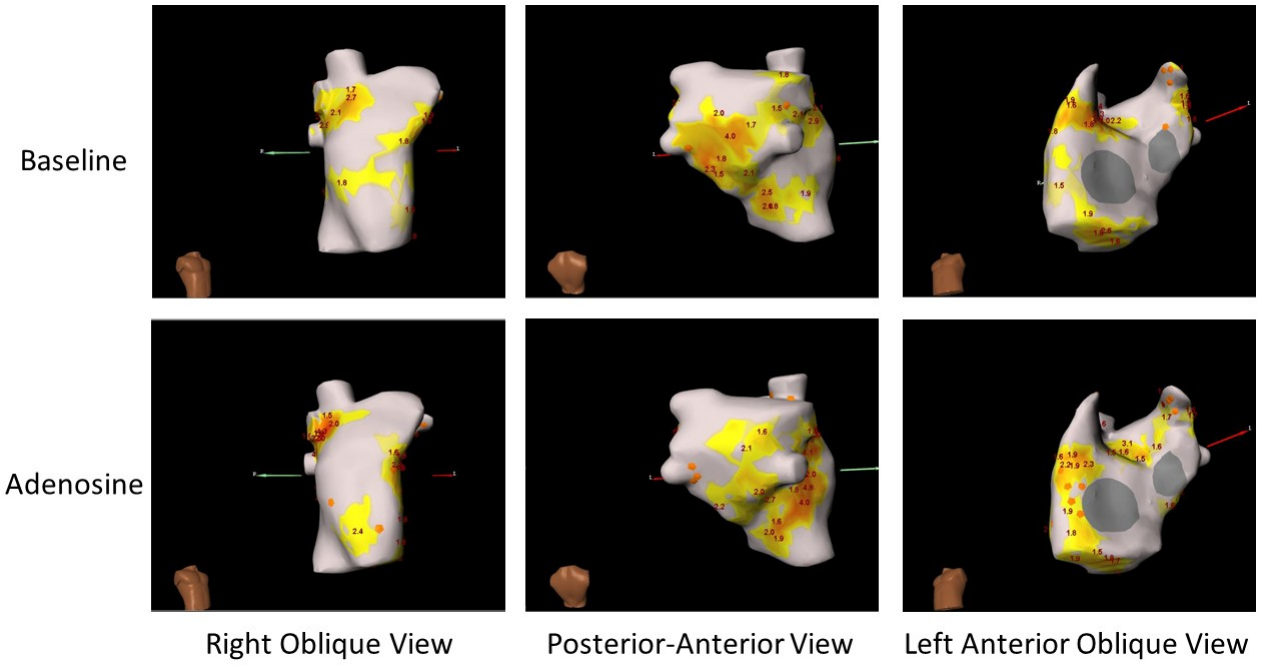
ECGI composites maps at baseline and with adenosine. ECGI Composite maps from the same patient at baseline and with Adenosine. In this figure the orange hexagons represent focal drivers and the yellow/orange areas are regions where rotational drivers have been detected. The numbers in the diagram represent the number of revolutions completed for each rotational PD occurrence in that region. the overall burden and distribution of PDs is very similar, but the proportion of PDs that are focal is arguable slightly increased in the maps following administration of adenosine.

There was a small but significant difference in the percentage of PDs that were focal with a higher percentage of focal PDs detected post adenosine (36.2 ± 15.2) compared to the baseline maps (32.2 ± 14.4; p < 0.001). Examples of maps pre and post adenosine are shown in Fig 4. On a regional basis there was a trend towards significance in the percentage of PDs that were focal at the PVs and PW post adenosine (55.2 ± 29.8) compared to the baseline maps (43.7 ± 27.8; p = 0.052). Excluding the PVs and PW, the percentage of PDs that were focal was higher following adenosine (30.7 ± 13.9) than on baseline maps (28.0 ± 15.8; p = 0.003).

### Contact mapping measurements

There was a significant reduction in LAA and RAA CL following the administration of adenosine: 165.6 ± 31.7 vs 148.3 ± 28.4; p = 0.003 and 176.7 ± 34.7 vs 149.9 ± 27.7; p < 0.001 (Table 4). There was a significant reduction in CL in the RAA 20.9 (6.9–41.9), compared to the LAA (7.0 (0.1–33.9); p = 0.030). This translated to a 4.9 (0.5–20.7) % change in the LAA and a 13.8 (4.4–24.6) % change in the RAA CL (p = 0.068).

**Table 4:** Cycle lengths at baseline and with adenosine.

	Baseline	Adenosine	P Value
Left Atrial Appendage (LAA)	165.6 ± 31.7	148.3 ± 28.4	0.003
Right Atrial Appendage (RAA)	176.7 ± 34.7	149.9 ± 27.7	<0.001

Values given as mean ± standard deviation or median (interquartile range).

#### Correlation between AF duration and left atrial dimensions and impact of adenosine

LA diameter showed a significant correlation with change in RAA CL with adenosine (r = − 0.389, p = 0.032), and a trend towards correlation with change in LAA CL (r = − 0.325, p = 0.070) (S1 Table). There was a trend towards correlation between LA diameter and impact of adenosine on PD burden (r = 0.253, p = 0.106), but not on PD distribution (r = − 0.093, p = 0.560). There was no correlation between AF duration and the impact of adenosine on any of these factors (all r < 0.1 and p > 0.10).

### Multivariate analysis

Outcomes of the multivariate analysis are included in S2–S5 Tables. The only factor remaining in the final model predicting an increase in PD burden was LA diameter which trended towards significance (OR 1.133, 95 CI 0.994–1.291, p = 0.062). Factors associated with an increase in PD distribution in the final model were male gender (OR 10.31, 95 CI 1.206–88.096, p = 0.033), age (OR 0.883, 95 CI 0.799–0.976, p = 0.015), diabetes mellitus (OR 10.274, 95 CI 0.762–138.579, p = 0.079) and ischaemic heart disease (OR 0.063, 95 CI 0.003–1.327, p = 0.075). There were no significant predictors of reduction in RAA CL although age (OR 1.096, 95 CI 0.984–1.222, p = 0.096), LA diameter (OR 0.877. 95 CI 0.755–1.018, p = 0.085) and ischaemic heart disease (OR 0.107, 95 CI 0.008–1.427, p = 0.091) trended towards significance. There were no significant predictors of reduction in LAA CL.

### Power calculations

There were limited data for *a priori* sample size estimation, so calculations were performed retrospectively to determine the power to detect clinically important changes. Calculations were performed for what were considered the two main parameters: total PD burden (the number of PD occurrences) and PD distribution (the number of segments on an 18 segment model of the atria harbouring drivers) using the data from baseline maps. We considered that a 15% change in either variable would have been considered clinically or biologically important. With a study population of 46 and assuming an α = 0.05, there was an 87.2% power to detect a 15% difference in PD burden and a 99.0% power to detect the same difference in PD distribution.

## Discussion

### Main findings

This is the first study to evaluate the impact of adenosine upon mechanisms sustaining persistent AF using both contact mapping and non-invasive mapping with the ECGI system. There was no significant effect of adenosine on the number of PDs observed or the distribution of PDs throughout the atria. There was no impact observed on the driver stability in terms of the number of consecutive cycles occurring. However, there was a small but significant increase in the proportion of drivers that were focal following administration of adenosine. Contact mapping confirmed a significant shortening of CL with adenosine that was more marked in the RAA than the LAA.

### Impact of adenosine upon AF mechanisms

The current study has demonstrated that although the ECGI system has demonstrated no impact of adenosine on driver burden, distribution, or stability, it did cause a small increase in the proportion of PDs that were focal which seemed evident to a similar extent at the PVs and posterior wall compared to the rest of the atria.

Adenosine causes a heterogenous reduction in atrial refractory periods which seems to affect the right atrium to a greater extent than the left [11]. Adenosine can also induce PV ectopy which has been proposed to occur due to increased autonomic output [10, 11, 14, 15]. Adenosine may trigger focal PDs through increasing automaticity caused by increased ganglionated plexi innervation or potentially via rebound sympathetic drive that has been shown to occur post adenosine administration [16].

Adenosine has previously been shown to increase dominant frequency of AF [17, 18]. There is also site-specific variation in the effect of adenosine which increases dominant frequency in the RA to a greater extent than is observed in the LA [11]. This is compatible with the findings of the current study, with adenosine causing a significant reduction in LAA and RAA CL, with the effect being greater in the RAA than the LAA. It is unclear what mediates this reduction in CL. The reduction in atrial refractory periods which are more marked in the RA than the LA could account for this. This is perhaps a more likely cause than the small increase in the proportion of PDs that were focal demonstrated in this study, since the total number of PDs was not affected.

#### Impact of adenosine in subgroups

The multivariate analyses and correlation studies suggest firstly that there is a heterogenous response to adenosine, but also that the impact on CL and impact on driver characteristics may differ. The trends taken together suggests a weak effect of increasing LA size being associated with an increase in PD burden with adenosine, whereas increasing LA size predicted less change in RAA CL. There was a trend towards female gender being associated with greater distribution of PDs with adenosine. Increasing age was associated with less impact of adenosine on PD distribution, but was associated with a greater impact on RAA CL. These sub-group analyses raise interesting questions about a heterogenous effect of adenosine, but the numbers may be too small to draw firm conclusions. This warrants further study.

### Clinical implications

These data have two important clinical implications. Firstly, that adenosine does impact on atrial physiology in terms of causing a small reduction in CL which is compatible with the shortening of action potential duration and refractory periods observed experimentally, and the increase in dominant frequency observed in humans [11, 18]. These changes were more pronounced in the right atrium than the left. However, analysis using the ECGI system did not demonstrate a great impact on driver mechanisms, other than a small increase in the proportion of drivers that were focal. Li et al., have suggested that expression of channels of the adenosine signalling pathway correlate to areas of localised drivers raising the question as to whether adenosine may have a role in potentially unmasking the location of AF drivers, particularly in the right atrium where these channels are highly expressed [15]. These data do not suggest a role for adenosine in highlighting drivers for ablation.

Secondly, the impact on driver burden and distribution assessed using the ECGI system was small, with no discernible change in most of the parameters measured. The only change detected was an increase in the proportion of PDs that were focal. Therefore, the use of adenosine to slow the ventricular rate in AF sufficiently for ECGI analysis (or arguably other non-contact mapping systems), the error introduced is small. Therefore, the use of adenosine in clinical cases and research studies is reasonable.

### Limitations

Although some analysis utilised contact electrograms, much of these data were derived using the ECGI system to identify rotational and focal activity. Although there is some data validating ECGI analysis, it is difficult to fully validate the system in terms of driver detection in AF since there is no universally accepted gold standard for comparison. It is accepted that not all PDs observed using the system are mechanistically important or real. Nevertheless, over a large group of patients any important effects of adenosine ought to have been detectable. It is noteworthy that different catheters were used to record electrograms in the LAA and RAA. Electrograms recorded in these locations are usually organised and the CL is un-ambiguous (as shown in Fig 3). Nevertheless, it is recognised that this introduces heterogeneity in how electrograms were recorded which could have impacted CL measurement.

## Conclusion

Adenosine caused a reduction in CL which was greater in the RAA than the LAA, compatible with previous data showing increased dominant frequency thought to be due to shortened refractory periods with adenosine which were more pronounced in the RA than the LA. This did not translate into an impact on driver burden, distribution or temporal stability. There was a small but significant increase in the proportion of PDs that were focal which could be compatible with the transient increase in autonomic drive and increased PV firing demonstrated by others. These observational data were largely acquired using the ECGI mapping system and further studies utilizing different mapping technologies are warranted. The impact of adenosine on ECGI mapping data was small though, suggesting that the use of adenosine during ECGI or non-contact mapping is unlikely to substantially impact the mapping data acquired.

## Supporting information

S1 Table Correlation between duration of AF or left atrial diameter and the impact of adenosine. Spearman’s correlation analysis was performed with a p < 0.05 taken to be significant.
(DOCX)

S2 Table Binary logistic regression analysis of factors predicting increase in PD burden following administration of adenosine. An 15% increase in PD burden following administration of adenosine was thought to be clinically significant and designated a positive response. A p < 0.05 was taken to be significant.
(DOCX)

S3 Table Binary logistic regression analysis of factors predicting increase in PD distribution following administration of adenosine. An 15% increase in PD distribution following administration of adenosine was thought to be clinically significant and designated a positive response. A p < 0.05 was taken to be significant.
(DOCX)

S4 Table Binary logistic regression analysis of factors predicting a decrease in right atrial appendage cycle length following administration of adenosine. A 15% decrease in RAA Cycle length following administration of adenosine was thought to be clinically significant and designated a positive response. A p < 0.05 was taken to be significant.
(DOCX)

S5 Table Binary logistic regression analysis of factors predicting a decrease in left atrial appendage cycle length following administration of adenosine. A 15% decrease in LAA Cycle length following administration of adenosine was thought to be clinically significant and designated a positive response. A p < 0.05 was taken to be significant.
(DOCX)

S1 Checklist TREND statement checklist. (PDF)

S1 File (DOCX)
