# Supplementary material for: Impact of adenosine on mechanisms sustaining persistent atrial fibrillation: Analysis of contact electrograms and non-invasive ECGI mapping data
Source: PLoS One. 2021 Mar 25;16(3):e0248951. doi: 10.1371/journal.pone.0248951 (PMC7993562; doi:10.1371/journal.pone.0248951)
Supplement: S1 Table — Spearman’s correlation analysis was performed with a p < 0.05 taken to be significant. (DOCX) [file pone.0248951.s001.docx]

**S1 Table. Correlation between duration of AF or left atrial diameter and the impact of Adenosine**

| **Factors** | **Atrial Fibrillation Duration** | **LA Diameter** |
| --- | --- | --- |
| Difference in LAA CL (Rho) | 0.015 | -0.325 |
| P Value | 0.932 | 0.070 |
| Difference in RAA CL (Rho) | 0.098 | -0.380 |
| P Value | 0.589 | 0.032 |
| Difference in PD Burden (Rho) | -0.011 | 0.253 |
| P value | 0.944 | 0.106 |
| Difference in PD distribution (Rho) | -0.119 | -0.093 |
| P Value | 0.431 | 0.560 |

Spearmans correlation analysis was performed with a p < 0.05 taken to be significant.
