# Supplementary material for: Impact of adenosine on mechanisms sustaining persistent atrial fibrillation: Analysis of contact electrograms and non-invasive ECGI mapping data
Source: PLoS One. 2021 Mar 25;16(3):e0248951. doi: 10.1371/journal.pone.0248951 (PMC7993562; doi:10.1371/journal.pone.0248951)
Supplement: S2 Table — An 15% increase in PD burden following administration of adenosine was thought to be clinically significant and designated a positive response. A p < 0.05 was taken to be significant. (DOCX) [file pone.0248951.s002.docx]

**S2 Table. Binary Logistic Regression analysis of factors predicting increase in PD Burden following administration of adenosine.**

| **Factor** | **Odds Ratio** | **95 % Confidence Interval** | **P Value** |
| --- | --- | --- | --- |
| Male gender | 1.644 | 0.230 – 11.748 | 0.620 |
| Age | 1.021 | 0.938 – 1.110 | 0.632 |
| LA Diameter | 1.151 | 0.998 – 1.326 | 0.053 |
| Hypertension | 1.234 | 0.187 – 8.161 | 0.827 |
| Diabetes Mellitus | 1.758 | 0.281 – 11.018 | 0.547 |
| Ischaemic Heart Disease | 0.568 | 0.049 – 6.571 | 0.651 |
| Duration of AF | 0.968 | 0.871 – 1.075 | 0.542 |

An 15 % increase in PD burden following administration of adenosine was thought to be clinically significant and designated a positive response. A p < 0.05 was taken to be significant.
