# Supplementary material for: Impact of adenosine on mechanisms sustaining persistent atrial fibrillation: Analysis of contact electrograms and non-invasive ECGI mapping data
Source: PLoS One. 2021 Mar 25;16(3):e0248951. doi: 10.1371/journal.pone.0248951 (PMC7993562; doi:10.1371/journal.pone.0248951)
Supplement: S3 Table — An 15% increase in PD distribution following administration of adenosine was thought to be clinically significant and designated a positive response. A p < 0.05 was taken to be significant. (DOCX) [file pone.0248951.s003.docx]

**S3 Table. Binary Logistic Regression analysis of factors predicting increase in PD distribution following administration of adenosine.**

| **Factor** | **Odds Ratio** | **95 % Confidence Interval** | **P Value** |
| --- | --- | --- | --- |
| Male gender | 10.679 | 0.699 – 163.267 | 0.089 |
| Age | 0.882 | 0.792 – 0.982 | 0.022 |
| LA Diameter | 0.986 | 0.839 – 1.158 | 0.860 |
| Hypertension | 1.238 | 0.104 – 14.792 | 0.866 |
| Diabetes Mellitus | 9.789 | 0.562 – 170.481 | 0.118 |
| Ischaemic Heart Disease | 0.052 | 0.002 – 1.540 | 0.087 |
| Duration of AF | 0.983 | 0.872 – 1.107 | 0.774 |

An 15 % increase in PD distribution following administration of adenosine was thought to be clinically significant and designated a positive response. A p < 0.05 was taken to be significant.
