# Supplementary material for: Impact of adenosine on mechanisms sustaining persistent atrial fibrillation: Analysis of contact electrograms and non-invasive ECGI mapping data
Source: PLoS One. 2021 Mar 25;16(3):e0248951. doi: 10.1371/journal.pone.0248951 (PMC7993562; doi:10.1371/journal.pone.0248951)
Supplement: S4 Table — A 15% decrease in RAA Cycle length following administration of adenosine was thought to be clinically significant and designated a positive response. A p < 0.05 was taken to be significant. (DOCX) [file pone.0248951.s004.docx]

| **Factor** | **Odds Ratio** | **95 % Confidence Interval** | **P Value** |
| --- | --- | --- | --- |
| Male gender | 0.115 | 0.004 – 3.732 | 0.223 |
| Age | 1.166 | 1.007 – 1.351 | 0.041 |
| LA Diameter | 0.770 | 0.588 – 1.009 | 0.058 |
| Hypertension | 0.257 | 0.013 – 4.906 | 0.367 |
| Diabetes Mellitus | 11.714 | 0.541 – 253.669 | 0.117 |
| Ischaemic Heart Disease | 0.136 | 0.004 – 4.620 | 0.268 |
| Duration of AF | 1.111 | 0.958 – 1.288 | 0.163 |

**S4- Table. Binary Logistic Regression analysis of factors predicting a decrease in Right Atrial Appendage Cycle Length following administration of adenosine.**

A 15 % decrease in RAA Cycle length following administration of adenosine was thought to be clinically significant and designated a positive response. A p < 0.05 was taken to be significant.
