# Supplementary material for: Impact of adenosine on mechanisms sustaining persistent atrial fibrillation: Analysis of contact electrograms and non-invasive ECGI mapping data
Source: PLoS One. 2021 Mar 25;16(3):e0248951. doi: 10.1371/journal.pone.0248951 (PMC7993562; doi:10.1371/journal.pone.0248951)
Supplement: S5 Table — A 15% decrease in LAA Cycle length following administration of adenosine was thought to be clinically significant and designated a positive response. A p < 0.05 was taken to be significant. (DOCX) [file pone.0248951.s005.docx]

**S5 Table. Binary Logistic Regression analysis of factors predicting a decrease in Left Atrial Appendage Cycle Length following administration of adenosine.**

| **Factor** | **Odds Ratio** | **95 % Confidence Interval** | **P Value** |
| --- | --- | --- | --- |
| Male gender | 1.329 | 0.103 - 17.180 | 0.828 |
| Age | 1.068 | 0.960 – 1.188 | 0.230 |
| LA Diameter | 0.965 | 0.817 – 1.140 | 0.678 |
| Hypertension | 2.077 | 0.183 – 23.568 | 0.555 |
| Diabetes Mellitus | 2.466 | 0.226 – 26.946 | 0.460 |
| Ischaemic Heart Disease | 0.292 | 0.014 – 6.035 | 0.426 |
| Duration of AF | 0.948 | 0.848 – 1.059 | 0.345 |

A 15 % decrease in LAA Cycle length following administration of adenosine was thought to be clinically significant and designated a positive response. A p < 0.05 was taken to be significant.
