# Supplementary material for: Impact of adenosine on mechanisms sustaining persistent atrial fibrillation: Analysis of contact electrograms and non-invasive ECGI mapping data
Source: PLoS One. 2021 Mar 25;16(3):e0248951. doi: 10.1371/journal.pone.0248951 (PMC7993562; doi:10.1371/journal.pone.0248951)
Supplement: S1 File — (DOCX) [file pone.0248951.s007.docx]

# ECG-I Mapping of Atrial Fibrillation to Optimise Patient Selection and Targeting of Ablation Therapy.

**Lay summary**

Atrial fibrillation (AF) is characterised by multiple reentry circuits in the top chambers of the heart (the atria). Catheter ablation is a treatment which tries to eliminate AF by cauterizing small patches of heart tissue in strategic locations. However, although ablation at certain locations seems to work for early AF, it is notoriously difficult to locate and target the areas driving established AF. The ECG-I is a system which involves wearing a jacket with many ECG electrodes to record electrical activity from the surface of the body. A CT scan then shows where these electrodes are relative to the atria, and computer modelling is used to reconstruct the movements of electricity on the surface of the heart and therefore where the areas driving AF are located. There are 2 arms to this study. The *‘Combined approach’* study arm will involve targeting the pulmonary veins (the standard ablation therapy for AF) followed by detailed mapping to look for evidence of drivers at the locations predicted by the ECG-I system and targeting of these areas to see if AF slows or terminates to the normal rhythm. These patients will then be followed up to look at the response. The *‘staged approach’* study arm will involve 2 phases. Phase 1 will involve ECG-I analysis followed by standard ablation targeting the pulmonary veins to see if activation patterns can predict who will respond to this quick low risk procedure. Those patients with recurrent AF after phase 1 will progress to phase 2 of this arm where they will have ablation targeting the drivers of AF directly based on ECG-I analysis and will be followed up to evaluate their response.

**Introduction**

Outcomes for catheter ablation of persistent AF are still lagging behind those for paroxysmal AF. Pulmonary vein isolation (PVI) alone seems sufficient to remove the trigger for the vast majority of patients with paroxysmal AF. However, in patients with persistent AF it is common for AF to continue after isolation of the pulmonary veins (PVs). The atria dilate and remodel structurally and electrically. Persistent AF then seems to be maintained by focal sources, whether rotors or sites of radial activation.^1, 2^ Although AF can sometimes be terminated by targeting surrogates for these drivers such as fractionated electrograms, this is imprecise and requires extensive ablation, and even then the mechanisms sustaining AF may not be interrupted. In fact, recent data suggests that current methods to ablate persistent AF beyond PVI such as ablation of fractionated electrograms and linear lesions are ineffective in terms of preventing recurrent arrhythmia.^3^

Termination of AF seems to be a marker of a good outcome.^4^ This may be partly a property of the patient’s substrate (i.e. less remodelled atria) and patients who have been in AF for a shorter duration and with less dilated atria are more likely to terminate to sinus rhythm during ablation and to remain free from arrhythmia subsequently.^4^ However, current methods to select patients likely to benefit from ablation based on such clinical characteristics or structural imaging have limited accuracy.^5^ Mapping studies have shown that patients with persistent AF who have higher frequency signals near the pulmonary vein ostia than the left atrial body are more likely to terminate to sinus rhythm with PVI alone and to maintain sinus rhythm subsequently.^6^ Similarly, these studies have suggested that patients undergoing conventional ablation procedures for persistent AF who have coincidental interruption of drivers have a far better long term outcome.^7^ These data suggest that the atrial substrate and consequent activation patterns maintaining AF are likely to determine the response to ablation in a given area. Therefore, rather than looking at surrogate factors to predict who will respond to AF ablation in general and PVI in particular, it may be possible to determine this more directly and accurately by panoramically mapping the atria.

It appears that a proportion of patients with persistent AF will maintain sinus rhythm long term after PVI alone.^3, 6, 8^ PVI can now be achieved quickly and safely using technologies such as the Cryoballoon.^9^ Identification of patients that are likely to respond to PVI alone is therefore of great interest as it (1) identifies patients that may respond to a conservative strategy, and (2) in the absence of an effective strategy beyond PVI may allow de-selection of patients unlikely to benefit from ablation at all. Although conventionally patients have been categorized according to whether AF is intermittent or how long it has been persistent for, a far more useful categorization for therapeutic purposes would be to identify those with AF driven by the PVs and who will respond to PVI, and those with non-PV driven AF. It is proposed that non-invasive mapping using the ECG-I might allow identification of certain activation patterns sustaining AF that will better respond to PVI, and just as importantly those that will not.

Non-invasive mapping has been used to characterize wavefront activation in AF. In AF that is still paroxysmal drivers seem to be located predominantly at the pulmonary vein ostia and the posterior wall,^10, 11^ whereas in persistent AF the drivers become more numerous and more spatially diverse.^2^ The AFACART study is a multicenter study that has demonstrated that ablation of persistent AF guided by non-invasive mapping of drivers using the ECG-I system can interrupt the mechanisms maintaining AF, thus terminating AF with relatively little ablation.^12^ It is conceivable that this may improve outcomes for some patients with persistent AF when performed in conjunction with PVI compared to PVI alone. However, at present it remains unclear who will respond to PVI alone and who is unlikely to respond to ablation at all, and hence the ideal population to target with such a strategy remains unclear.

The *‘staged approach’* arm of the study will evaluate the clinical utility of the ECG-I in this context. For phase 1 of this study we propose to map a large cohort of patients with persistent AF of up to 24 months duration non-invasively using the ECG-I. All patients in the cohort will then undergo PVI alone. If patients do not revert to sinus rhythm following ablation they will be DC cardioverted. Patients will then be followed up for 1 year to determine their response to ablation. We hypothesize that the presence of drivers exclusively at the pulmonary vein antra or posterior wall predicts a higher rate of termination to sinus rhythm following PVI and a better long term outcome with this conservative strategy compared to patients with a wider distribution of drivers. Furthermore, a greater proportion of drivers located at the PV ostia and posterior wall (according to the aggregated driver density map) will predict a graded increase in the success rate with PVI alone.

Patients will undergo repeat mapping with the ECG-I immediately following PVI but prior to DC cardioversion to determine how PVI has impacted on the driver maps acutely. It will be important to determine (1) whether there are fewer drivers after elimination of those near the PVs (or whether new regions have simply become active in their place), and (2) whether those drivers outside the PVs are consistent after PV isolation. This will provide a proof of concept for the rationale for targeting drivers at all.

Phase 2 of the study will involve those patients with recurrent AF following their index procedure (PVI alone as per phase 1). A repeat study will be performed with the ECG-I to determine whether the same un-interrupted drivers are responsible for maintaining AF, or whether the mechanisms of recurrence are different and adaptive. These patients with recurrent AF despite PVI alone will then undergo repeat ablation involving completion of PVI if pulmonary veins have reconnected, followed by direct targeting of drivers using the ECG-I system. This will be targeting drivers in order of those most active according to the aggregated driver density map, aiming for termination to sinus rhythm. If sinus rhythm is achieved then that will be regarded as an end-point and no further ablation delivered. If AF terminates to AT this will be mapped and ablated. If AF persists the patient will be DC cardioverted and will not receive a ‘step-wise style’ procedure. Patients will then be followed up for 1 year to determine their response to ablation. This will allow us to determine the staged procedure outcome using a conservative approach of PVI initially followed by direct targeting of drivers for those with recurrent arrhythmia in an unselected cohort. This will also better define the cohort of patients with persistent AF who are unlikely to respond to ablation at all.

A separate cohort of 50 patients (meeting the recruitment criteria for phase 1) will be mapped non-invasively using the ECG-I prior to their ablation procedure as part of the ‘*combined approach’* study arm. Following PVI patients will undergo detailed electroanatomic mapping so as to validate the ECG-I system by demonstrating the existence of electrical activity consistent with driver regions using contact electrograms at sites where drivers are predicted by the ECG-I system. We will also study physiological correlates of drivers in terms of scar, zones of slow conduction, and other phenomena so as to improve our understanding of AF generally. This cohort will then undergo targeting of drivers at the index procedure looking at the proportion of patients in whom AF terminates or organizes with targeting of these regions alone. Patients remaining in AF will then be DC cardioverted. Those patients organising to AT will have this mapped and ablated also. Otherwise patients will not cross over to a conventional stepwise approach). They will then be followed up for 1 year to determine the success of this approach.

**Objectives**

The hypotheses for the four studies that comprise this project are as follows:

**Hypothesis 1: Staged approach arm - phase 1**

The presence of drivers exclusively at the pulmonary vein antra or posterior wall predicts a higher rate of termination to sinus rhythm following PVI and a better long term outcome compared to patients with a wider distribution of drivers, as predicted by non-invasive mapping.

To prove this we propose an international multicenter observational cohort study recruiting 100 patients with persistent AF (phase 1 above). All patients will undergo mapping with the ECG-I prior to PVI using the cryoballoon. Patients will then be followed up for 1 year to determine the response to ablation (Figure 1).

Phase 1 has the novel objectives:

1. To determine whether there is an activation pattern in persistent AF discernible from non-invasive mapping that can predict the response to PVI alone?
2. If so, is this independently predictive once other factors such as duration of persistent AF, left atrial volume are taken into account?
3. If so, would the use of this system as a screening tool be cost effective to perform routinely?

**Hypothesis 2 - Staged approach arm - phase 1, remapping post PVI**

PVI organizes AF by eliminating drivers

To prove this we will re-map 30 patients with the ECG-I after PVI but before DC cardioversion. This study has the following objectives:

1. To broadly explore the impact of PVI on activation patterns in AF.
2. To show that elimination of drivers acutely organizes AF, i.e. these drivers are not simply replaced by others.
3. To show that drivers outside the PV antra remain consistent after PVI and hence that these may be worth targeting acutely after PVI.

**Hypothesis 3: Staged approach arm - phase 2**

For patients not responding to PVI at the index procedure, a repeat procedure involving PV re-isolation and targeting of drivers guided by the ECG-I will terminate AF and result in subsequent freedom from AF in a significant proportion.

Phase 2 will have the following novel objectives:

1. To explore to impact of ablating driver regions in a cohort who have failed to respond to PVI (for whom there is currently no strategy proven to be effective), both acutely and long term.
2. To report the procedural metrics and success rates following phase 1 and 2 as a conservative strategy with 2 quick and low risk procedures.

**Hypothesis 4: Combined approach study arm**

Sites of drivers predicted by the ECG-I system correlate with activation patterns identified by endocardial mapping, and ablation of these following PVI will result in termination of AF acutely and freedom from AF long term in a significant proportion.

This validation phase will have the following novel objectives:

1. To validate the ECG-I system in it’s identification of drivers.
2. To explore physiological correlates of drivers to improve our understanding of AF.
3. To demonstrate the impact of targeting driver sites both acutely and long term.

**Study Methods**

**Inclusions:**

Patients with persistent AF (i.e. episodes of AF that are continuous for > 1 week or have required DC cardioversion)^13^ willing for ablation.

**Exclusions:**

• Persistent AF diagnosed > 2 years ago

• LA diameter > 5 cm

• Severe left ventricular impairment (EF < 40%)

• NYHA class 3 or 4 heart failure

• Known hypertrophic cardiomyopathy, cardiac sarcoid or ARVC.

• Known inherited arrhythmia such as Brugada or long QT syndromes

• Valvular disease that is more than moderate

• History of valve replacement (metallic or tissue)

• History of congenital heart disease (other than patent foramen ovale)

• Previous left atrial ablation (percutaneous or surgical)

• Cardiac surgery or PCI within the last 3 months.

• Myocardial infarction or unstable angina within the last 3 months.

• Unwillingness for ablation

• Unwillingness to be involved in study

• Suspected reversible cause of AF

• Any other contraindication to catheter ablation

• Age < 18 yrs or > 80 years

• Pregnancy

• Morbid obesity (defined as BMI >40)

• Any other medical problem likely to cause death within the next 18 months

**
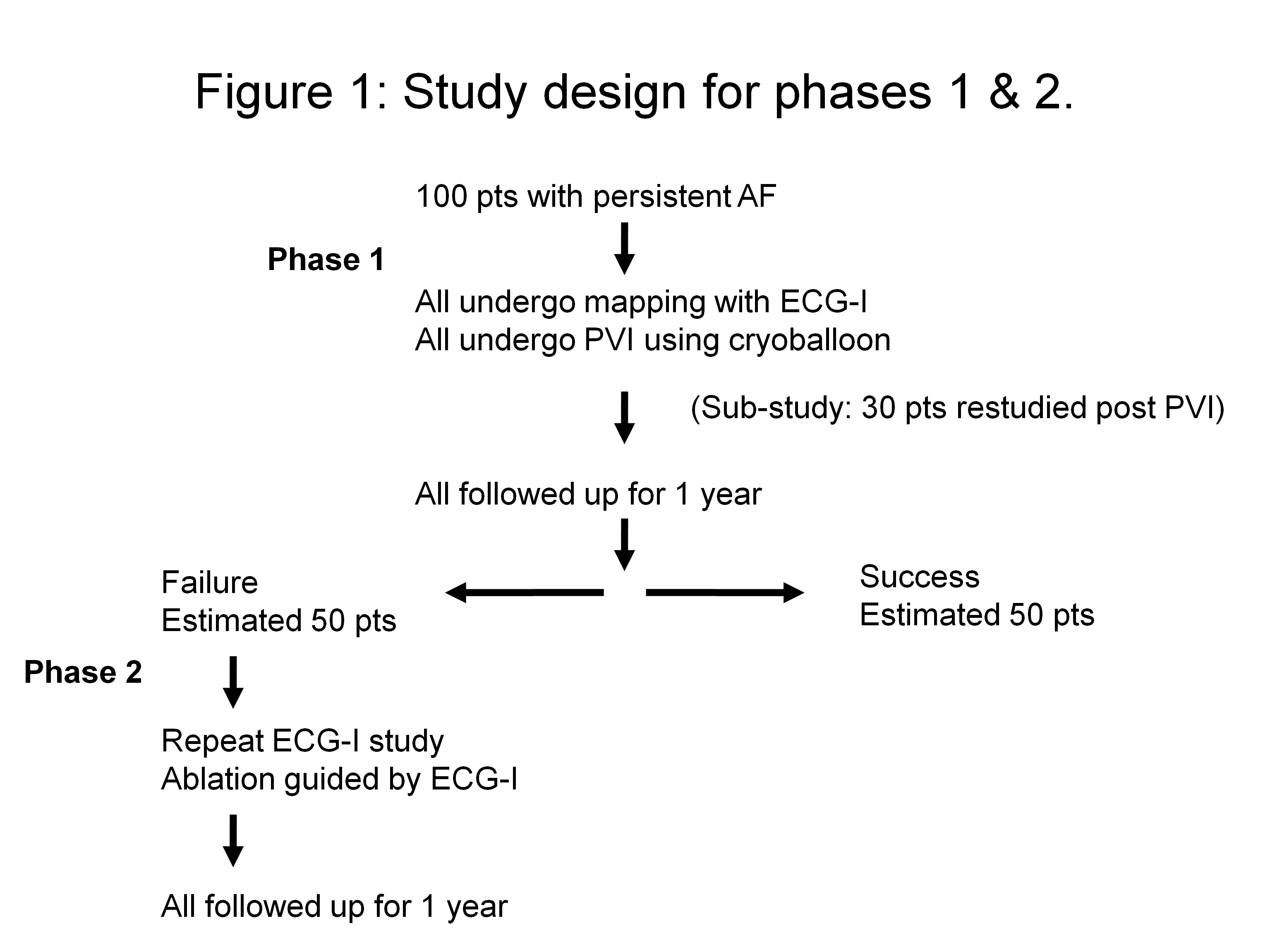
**

**Imaging and non-invasive mapping methods**

The acquisition of cardiac signals from the patient’s torso and computational reconstruction of non-invasive maps have been described previously.^13^ Briefly, patients will wear the proprietary vest of 252 electrodes which is connected to the non-invasive imaging system that records cardiac electrical activity. The location of electrodes on the torso and their location relative to the heart chamber is obtained by computed tomography (CT) scan. The 3D chamber geometries (atrial and/or ventricles) will be reconstructed and their position relative to the body surface electrodes on the torso visualized. The system will then compute activation maps from torso signals using mathematical reconstruction algorithms. An aggregated driver-density map will then be constructed for each patient, which summates all the drivers recorded and projects them onto the patient’s biatrial geometry.

**Catheter ablation: phase 1 of the staged approach study arm**

Procedures will be performed on oral anticoagulation under moderate sedation or general anaesthesia. Catheters will be introduced through the right femoral vein. The target activated clotting time will be 300 - 350s with boluses of un-fractionated heparin.

Two sheaths will be placed in the right femoral vein. A single transseptal puncture will be performed and a sheath advanced into the left atrium. The Cryoballoon (Arctic Front Advance or subsequent generation, Medtronic Inc, Minneapolis, USA) and the Achieve mapping catheter (or subsequent generation of this catheter; Medtronic Inc, Minneapolis, USA) will be used for ablation. After applying the cryoballoon to the PV ostium, contrast will be injected into the PV to show ostial occlusion. Target cryoballoon temperatures will be ≤ -40^0^C and a single successful application of 180 seconds will be applied to each PV ostium.

During ablation of the right PVs, a catheter will be moved to the SVC and used to pace the phrenic nerve, so that cryoablation can be halted if incipient phrenic nerve injury is suspected.

If patients remain in AF/AT following isolation of all PVs they will be cardioverted with a DC shock. The only exception will be termination to typical right atrial flutter in which case a cavotricuspid isthmus line will be ablated with an irrigated ablation catheter.

**Follow up**

Patients will be discharged as soon as they are well and ambulant. Anti-arrhythmic drug therapy will be continued for up to 3 months then stopped if patients are free from atrial arrhythmias. DC cardioversion will be permitted for persistent arrhythmia within this 3 month blanking period. Patients with ongoing arrhythmia at 3 months will be offered a repeat ablation at that time (and counted as failure following their first ablation). Otherwise, arrhythmia that settles within this 3 month blanking period will not be counted as failure.

All patients will be followed up at 3, 6, 9 and 12 months following their procedure. Monitoring for asymptomatic arrhythmia will also be performed as per current guidelines:^14^ a 24 hour period of ambulatory monitoring will be recorded at 6 and 12 months, and in the event of recurrent symptoms between appointments there will be open access for further review and monitoring with an event recorder.

All patients will undergo quality of life assessment at baseline and final follow up to assess impact on quality of life and therefore allow calculation of cost benefit.

Those who fail their first ablation procedure within the year of the study will continue into phase 2 of the study. Following their repeat procedure guided by ECG-I they will restart their follow up to allow assessment of their 1 year success after a repeat procedure.

**Catheter ablation: phase 2 of the staged approach study arm**

Peri-operative management will be identical to that described above for phase 1. Patients will undergo their repeat procedure guided by a 3D mapping system using contact force sensing catheters as is usual care. PVs will be re-isolated where necessary. Drivers will then be targeted in order of those most active according to the aggregated driver density map, aiming for termination to sinus rhythm. If sinus rhythm is achieved then that will be regarded as an end-point and no further ablation delivered. If AF terminates to AT this will be mapped and ablated. If AF persists after ablation of drivers, the patient will be DC cardioverted and will not receive a ‘step-wise style’ procedure. Patients will be followed up as per the description above.

**Mapping study**

Patients will be studied with the ECG-I system pre-operatively. Again, peri-operative management will be identical to that described above for phase 1. Patients will undergo their procedure guided by a 3D mapping system using contact force sensing catheters and multipolar mapping catheters as is usual care. Mapping will focus on identification of driver sites corresponding to those identified by the ECG-I system. Patients will then undergo targeted ablation at these driver sites as describe in phase 2 above. Rates of AF cycle length prolongation and termination will be recorded. All patients will then undergo PV isolation. If AF terminates to AT this will be mapped and ablated. If AF persists after ablation of drivers, the patient will be DC cardioverted and will not receive a ‘step-wise style’ procedure. Patients will be followed up as per the description above.

**Study End Points**

The primary endpoint of the study will be whether the presence of drivers exclusively at the pulmonary vein antra and posterior wall predicts success rate at 1 year following a single procedure off anti-arrhythmic drugs. Success will be defined as freedom from documented AF/AT lasting ≥ 30s (whether symptomatic or not) following a 3 month blanking period, as per current guidelines.^14^ The use of anti-arrhythmic drugs after the 3 month blanking period will also be counted as failure.

Secondary endpoints will be:

**The staged approach arm - phase 1**

- Whether the presence of drivers exclusively at the pulmonary vein antra and posterior wall **independently** predicts success rate at 1 year on multivariate analysis (including patient age, gender, time since diagnosis of persistent AF, left atrial volume).
- A cost efficacy analysis of treatment which will include the cost of the procedure and any procedure or arrhythmia related admissions within the year of the study and quality of life data at 1 year to determine a cost effectiveness analysis. If the ECG-I system determines an activation pattern which is independently predictive of response to ablation, then health economic modeling will be used to determine the cost effectiveness of routinely using the ECG-I system at baseline with a view to de-select those likely to be non-responders.
- The crude single procedure success rate for cryoballoon PVI in persistent AF (<1 yr duration)
- The crude single procedure success rate for cryoballoon PVI in long standing persistent AF (>1 yr duration)
- The procedure time and fluoroscopy times for cryoballoon PVI in persistent AF
- The major complication rates for cryoballoon PVI in persistent AF.

**Phase 1 sub-study: acute effects of PVI**

- The number of new drivers emerging after elimination of PV/posterior wall drivers.
- The proportion of drivers outside the PV antra/posterior wall that remain consistent after PVI.

**The staged approach arm - phase 2**

- For those with recurrent AF, correlation between original driver sites and those on repeat ECG-I study.
- For those with recurrent AF despite PVI, is there a pattern (or number) of drivers discernible on non-invasive mapping that determines in which patients AF will terminate and result in freedom from AF subsequently
- The success rate at 1 year with a staged approach, involving a conservative strategy of PVI alone followed by direct targeting of drivers at repeat procedures.
- The cost efficacy and cost effectiveness of this approach.
- The cost effectiveness of performing the ECG-I routinely in patients with persistent AF who have failed PVI with a view towards de-selecting those with an activation pattern associated with failure.
- The proportion of patients terminating to sinus rhythm during ablation with targeting of drivers.
- Complication rate
- Procedure time
- Fluoroscopy time

Combined approach: mapping study

- Confirmation that sites identified as drivers by ECG-I correlate with plausible activation patterns on endocardial mapping (at least intermittently).
- Correlation of drivers with sites of abnormal pathophysiology such as low voltage, or other phenomena such as high wall stress,^15^ or ganglionated plexi innervation as described previously.^16^
- The proportion of patients terminating to sinus rhythm with PV isolation and targeting of drivers.
- The proportion of patients remaining free from AF at 1 year with targeting of drivers and PVI alone.

Frequency analysis sub-study

30second electrograms will be recorded from the left and right atrial appendages, the proximal coronary sinus, and the PVs in all patients (both ‘staged’ and ‘combined’ arms) to allow validation of the ECG-I system in terms of determining local AF cycle length and dominant frequency. The ECG-I mapping data will then be used to examine the hypothesis that shorter cycle length and higher dominant frequency at the PV ostia compared to rest of the atria predicts a higher success rate with a PVI alone strategy (staged arm).

Sample size estimation

There are no methods to determine sample size for such a study and no real data upon which to base them. Recruitment of 100 patients is both achievable and should allow ample power to detect the relationships described in the primary and secondary end-points. The 50 patients in the validation study ought to be ample for these purposes also.

**Expected value of results**

There is a wealth of information that should come from this programme. If the ECG-I can be used to predict who will respond to PVI this could revolutionalize selection for ablation. Furthermore this study should pave the way for predicting who might benefit from ablation guided by the ECG-I. It will also provide some provisional data on the impact of ECG-I guided ablation in patients who have not responded to PVI – a population in whom there are currently no treatment options that have proven efficacy.

Even if it emerges that the ECG-I is not very useful in this regard, this study will still provide useful data on the ablation of persistent AF using the cryoballoon.

**Feasibility of Study**

The Barts Heart Centre performs approximately 300-400 persistent AF ablations per year, at least half of whom would meet the recruitment criteria. We would therefore be able to complete recruitment alone within 12 months. Nevertheless other centres may be added to assist with recruitment and make this a multicenter study.

**Oversight**

The study will be administered by the Barts Heart Centre Arrhythmia Research Unit. The safety and efficacy of the approaches in phase 1 and phase 2 will be reviewed by an independent data safety monitoring committee biannually. It is not anticipated that the cohort should be exposed to any additional risk.

**Justification of Expenses**

The funding sought is for the additional monitoring and follow up required as part of a clinical trial, for administrative expenses, and for the salary of a both a nurse and a physiologist to support these studies. The application of the ECG-I jacket is time consuming and the acquisition of the maps requires expertise best suited to a dedicated physiologist to ensure optimal acquisition of the data. Although we have some funds for the ECG-I system, we will also require funding for the additional imaging and for the ECG-I system consumables.

Reference List

(1) Narayan SM, Krummen DE, Shivkumar K, Clopton P, Rappel WJ, Miller JM. Treatment of Atrial Fibrillation by the Ablation of Localized Sources: CONFIRM (Conventional Ablation for Atrial Fibrillation With or Without Focal Impulse and Rotor Modulation) Trial. *J Am Coll Cardiol* 2012 August 14;60(7):628-36.

(2) Haissaguerre M, Hocini M, Denis A, Shah AJ, Komatsu Y, Yamashita S, Daly M, Amraoui S, Zellerhoff S, Picat MQ, Quotb A, Jesel L, Lim H, Ploux S, Bordachar P, Attuel G, Meillet V, Ritter P, Derval N, Sacher F, Bernus O, Cochet H, Jais P, Dubois R. Driver domains in persistent atrial fibrillation. *Circulation* 2014 August 12;130(7):530-8.

(3) Verma A, Jiang CY, Betts TR, Chen J, Deisenhofer I, Mantovan R, MacLe L, Morillo CA, Haverkamp W, Weerasooriya R, Albenque JP, Nardi S, Menardi E, Novak P, Sanders P. Approaches to catheter ablation for persistent atrial fibrillation. *N Engl J Med* 2015 May 7;372(19):1812-22.

(4) O'Neill MD, Wright M, Knecht S, Jais P, Hocini M, Takahashi Y, Jonsson A, Sacher F, Matsuo S, Lim KT, Arantes L, Derval N, Lellouche N, Nault I, Bordachar P, Clementy J, Haissaguerre M. Long-term follow-up of persistent atrial fibrillation ablation using termination as a procedural endpoint. *Eur Heart J* 2009 May;30(9):1105-12.

(5) Marrouche NF, Wilber D, Hindricks G, Jais P, Akoum N, Marchlinski F, Kholmovski E, Burgon N, Hu N, Mont L, Deneke T, Duytschaever M, Neumann T, Mansour M, Mahnkopf C, Herweg B, Daoud E, Wissner E, Bansmann P, Brachmann J. Association of atrial tissue fibrosis identified by delayed enhancement MRI and atrial fibrillation catheter ablation: the DECAAF study. *JAMA* 2014 February 5;311(5):498-506.

(6) Pascale P, Shah AJ, Roten L, Scherr D, Komatsu Y, Ramoul K, Daly M, Denis A, Derval N, Sacher F, Hocini M, Jais P, Haissaguerre M. Pulmonary veins to left atrium cycle length gradient predicts procedural and clinical outcomes of persistent atrial fibrillation ablation. *Circ Arrhythm Electrophysiol* 2014 June;7(3):473-82.

(7) Narayan SM, Krummen DE, Clopton P, Shivkumar K, Miller JM. Direct or coincidental elimination of stable rotors or focal sources may explain successful atrial fibrillation ablation: on-treatment analysis of the CONFIRM trial (Conventional ablation for AF with or without focal impulse and rotor modulation). *J Am Coll Cardiol* 2013 July 9;62(2):138-47.

(8) Tilz RR, Rillig A, Thum AM, Arya A, Wohlmuth P, Metzner A, Mathew S, Yoshiga Y, Wissner E, Kuck KH, Ouyang F. Catheter ablation of long-standing persistent atrial fibrillation: 5-year outcomes of the Hamburg Sequential Ablation Strategy. *J Am Coll Cardiol* 2012 November 6;60(19):1921-9.

(9) Ciconte G, Ottaviano L, de AC, Baltogiannis G, Conte G, Sieira J, Di GG, Saitoh Y, Irfan G, Mugnai G, Storti C, Montenero AS, Chierchia GB, Brugada P. Pulmonary vein isolation as index procedure for persistent atrial fibrillation: One-year clinical outcome after ablation using the second-generation cryoballoon. *Heart Rhythm* 2015 January;12(1):60-6.

(10) Haissaguerre M, Hocini M, Shah AJ, Derval N, Sacher F, Jais P, Dubois R. Noninvasive panoramic mapping of human atrial fibrillation mechanisms: a feasibility report. *J Cardiovasc Electrophysiol* 2013 June;24(6):711-7.

(11) Cuculich PS, Wang Y, Lindsay BD, Faddis MN, Schuessler RB, Damiano RJ, Jr., Li L, Rudy Y. Noninvasive characterization of epicardial activation in humans with diverse atrial fibrillation patterns. *Circulation* 2010 October 5;122(14):1364-72.

(12) https://clinicaltrials.gov/ct2/show/NCT02113761. 2015.

Ref Type: Online Source

(13) Oster HS, Taccardi B, Lux RL, Ershler PR, Rudy Y. Noninvasive electrocardiographic imaging: reconstruction of epicardial potentials, electrograms, and isochrones and localization of single and multiple electrocardiac events. *Circulation* 1997 August 5;96(3):1012-24.

(14) Calkins H, Kuck KH, Cappato R, Brugada J, Camm AJ, Chen SA, Crijns HJ, Damiano RJ, Jr., Davies DW, DiMarco J, Edgerton J, Ellenbogen K, Ezekowitz MD, Haines DE, Haissaguerre M, Hindricks G, Iesaka Y, Jackman W, Jalife J, Jais P, Kalman J, Keane D, Kim YH, Kirchhof P, Klein G, Kottkamp H, Kumagai K, Lindsay BD, Mansour M, Marchlinski FE, McCarthy PM, Mont JL, Morady F, Nademanee K, Nakagawa H, Natale A, Nattel S, Packer DL, Pappone C, Prystowsky E, Raviele A, Reddy V, Ruskin JN, Shemin RJ, Tsao HM, Wilber D. 2012 HRS/EHRA/ECAS expert consensus statement on catheter and surgical ablation of atrial fibrillation: recommendations for patient selection, procedural techniques, patient management and follow-up, definitions, endpoints, and research trial design: a report of the Heart Rhythm Society (HRS) Task Force on Catheter and Surgical Ablation of Atrial Fibrillation. Developed in partnership with the European Heart Rhythm Association (EHRA), a registered branch of the European Society of Cardiology (ESC) and the European Cardiac Arrhythmia Society (ECAS); and in collaboration with the American College of Cardiology (ACC), American Heart Association (AHA), the Asia Pacific Heart Rhythm Society (APHRS), and the Society of Thoracic Surgeons (STS). Endorsed by the governing bodies of the American College of Cardiology Foundation, the American Heart Association, the European Cardiac Arrhythmia Society, the European Heart Rhythm Association, the Society of Thoracic Surgeons, the Asia Pacific Heart Rhythm Society, and the Heart Rhythm Society. *Heart Rhythm* 2012 April;9(4):632-96.

(15) Hunter RJ, Liu Y, Lu Y, Wang W, Schilling RJ. Left atrial wall stress distribution and its relationship to electrophysiologic remodeling in persistent atrial fibrillation. *Circ Arrhythm Electrophysiol* 2012 April;5(2):351-60.

(16) Lim PB, Malcolme-Lawes LC, Stuber T, Kojodjojo P, Wright IJ, Francis DP, Wyn DD, Peters NS, Kanagaratnam P. Stimulation of the intrinsic cardiac autonomic nervous system results in a gradient of fibrillatory cycle length shortening across the atria during atrial fibrillation in humans. *J Cardiovasc Electrophysiol* 2011 November;22(11):1224-31.
